# Triple Combination Antiviral Drug (TCAD) Composed of Amantadine, Oseltamivir, and Ribavirin Impedes the Selection of Drug-Resistant Influenza A Virus

**DOI:** 10.1371/journal.pone.0029778

**Published:** 2011-12-29

**Authors:** Justin D. Hoopes, Elizabeth M. Driebe, Erin Kelley, David M. Engelthaler, Paul S. Keim, Alan S. Perelson, Libin Rong, Gregory T. Went, Jack T. Nguyen

**Affiliations:** 1 Adamas Pharmaceuticals, Inc., Emeryville, California, United States of America; 2 Translational Genomics Research Institute, Flagstaff, Arizona, United States of America; 3 Los Alamos National Laboratory, Los Alamos, New Mexico, United States of America; 4 Oakland University, Rochester, Michigan, United States of America; Centers for Disease Control and Prevention, United States of America

## Abstract

Widespread resistance among circulating influenza A strains to at least one of the anti-influenza drugs is a major public health concern. A triple combination antiviral drug (TCAD) regimen comprised of amantadine, oseltamivir, and ribavirin has been shown to have synergistic and broad spectrum activity against influenza A strains, including drug resistant strains. Here, we used mathematical modeling along with three different experimental approaches to understand the effects of single agents, double combinations, and the TCAD regimen on resistance in influenza *in vitro*, including: 1) serial passage at constant drug concentrations, 2) serial passage at escalating drug concentrations, and 3) evaluation of the contribution of each component of the TCAD regimen to the suppression of resistance. Consistent with the modeling which demonstrated that three drugs were required to suppress the emergence of resistance in influenza A, treatment with the TCAD regimen resulted in the sustained suppression of drug resistant viruses, whereas treatment with amantadine alone or the amantadine-oseltamivir double combination led to the rapid selection of resistant variants which comprised ∼100% of the population. Furthermore, the TCAD regimen imposed a high genetic barrier to resistance, requiring multiple mutations in order to escape the effects of all the drugs in the regimen. Finally, we demonstrate that each drug in the TCAD regimen made a significant contribution to the suppression of virus breakthrough and resistance at clinically achievable concentrations. Taken together, these data demonstrate that the TCAD regimen was superior to double combinations and single agents at suppressing resistance, and that three drugs at a minimum were required to impede the selection of drug resistant variants in influenza A virus. The use of mathematical modeling with multiple experimental designs and molecular readouts to evaluate and optimize combination drug regimens for the suppression of resistance may be broadly applicable to other infectious diseases.

## Introduction

Combination drug therapies are well established as standard of care for the treatment of infection by rapidly mutating viruses such as HIV [1], where resistance arises rapidly to single agents and is associated with treatment failure [2]. Highly active antiretroviral therapy consisting of combinations of three or four reverse transcriptase inhibitors and protease inhibitors has transformed AIDS into a chronic and manageable disease from a fatal one, often extending the lifespan of patients by decades [3]. The lessons learned in HIV were that a combination of drugs with different mechanisms of action was required for maximal suppression of replication, resulting in sustained virologic response and reduced rate of resistance [4], [5]. More recently, the development of combination regimens for the treatment of hepatitis C and hepatitis B infections demonstrate that the findings from years of extensive clinical research in HIV may be applicable to other viral diseases as well [6], including influenza.

Currently there are only two classes of drugs approved for the treatment of influenza, the neuraminidase inhibitors (NAIs; oseltamivir and zanamivir) and the M2 channel inhibitors (amantadine and rimantadine). The 2009 influenza pandemic highlights the urgent need for new and more effective therapy, and the emergence of resistance to both drug classes among circulating influenza A strains has become a major public health concern. In the 2009–2010 season, 100% of tested H3N2 and 99.8% of the pandemic 2009 H1N1 subtypes were resistant to M2 channel inhibitors [7], and nearly 100% of the circulating seasonal H1N1 viruses tested in the 2008–2009 season were resistant to oseltamivir [8]. In 2009, the seasonal H1N1 viruses were replaced by the pandemic 2009 H1N1 viruses which at this time remain susceptible to oseltamivir [9]. While resistance to zanamivir has seldom been detected in the clinic, zanamivir resistant variants have been generated in cell culture [10], [11], [12], [13].

Treatment with antiviral drugs as single agents may be a contributing cause to the rise in resistance among influenza viruses. Oseltamivir-resistant variants have been induced in patients receiving treatment or chemoprophylaxis with oseltamivir [14], [15], although oseltamivir-resistant seasonal H1N1 viruses have also arisen in the absence of antiviral drug selection pressure [16], [17]. Given the widespread resistance patterns already existing among influenza strains, there is concern that monotherapy with antiviral drugs could lead to the development of dual-resistant viruses. Multidrug-resistant H3N2 influenza [18] and 2009 H1N1 viruses [19], [20] have been isolated from immunocompromised patients. Because current treatment options are limited for dual-resistant influenza viruses, continued surveillance of antiviral drug resistance patterns and the development of new antiviral strategies are of crucial importance for effective management of influenza virus infections.

A triple combination antiviral drug (TCAD) regimen comprised of amantadine (AMT), oseltamivir (OSC), and ribavirin (RBV) may be a viable therapeutic option that could address existing and emerging resistance in influenza A. In previous studies, TCAD has been shown to have broad spectrum activity and was synergistic against susceptible and resistant influenza strains [21], [22]. Importantly, the synergy of the TCAD regimen was greater than any double antiviral drug combination, and AMT and OSC contributed to the synergy of the TCAD regimen at concentrations that are clinically achievable against amantadine- and oseltamivir-resistant virus strains, respectively [21]. However, the effect of TCAD on the emergence of resistance has not been demonstrated to date. Our initial hypothesis was that a TCAD regimen incorporating drugs with three different mechanisms of actions is required to effectively reduce the levels of resistance compared to single and dual-drug therapies. In this paper, we report modeling results that motivated this effort and on the effects of TCAD on resistance *in vitro* by three different experimental methods: serial passage at constant drug concentrations, serial passage at increasing antiviral drug concentrations, and evaluating the contribution of each component of the TCAD regimen to the suppression of resistance. We show by all three methods that the TCAD regimen significantly suppressed the breakthrough of drug resistant virus *in vitro* and imposed a high genetic barrier to resistance compared to single and dual-drug therapies.

## Results

### Probability of developing resistance to 1-, 2-, or 3-drug regimens for influenza A virus

To determine from first principles the number of drugs required in a regimen to durably suppress the emergence of resistance in influenza A viruses, we calculated the probability of generating all possible 1-, 2-, or 3-base changes in the influenza A genome during the course of an acute infection in healthy individuals that we assume was initiated by a drug sensitive virus. We make a number of simplifying assumptions that affect the precise values of the probabilities we calculate but which do not affect the general conclusions that we reach. The first assumption was that 1-, 2-, or 3-base changes were required to develop resistance to 1, 2, or 3 drugs applied simultaneously, given that each drug acted on a different target and that a single base change could generate resistance to each drug. If influenza variants carrying all possible single-base changes were generated during the course of an infection, then there would be included among the population all possible drug resistant variants that require only a single nucleotide substitution to generate resistance. However, if only 1% of all possible variants were generated, then there would be a 1% chance that any particular drug resistant variant would be generated. In this calculation, all base changes are considered to be equally probable, conforming to the simplifying assumption that was made to estimate the genetic barrier needed for effective antiretroviral therapy against HIV infection [23]. Biases in either the gene in which the mutation occurs (e.g. M2 or NA) or the sites within these genes are not included in this first model. However, by using a mutation rate that is 10-fold lower than some reported values [24], [25], we are implicitly correcting for the possibility that some positions could be 10-fold more mutable. As shown in Table 1, all possible 1-base changes are expected to be generated during the course of an acute infection for H3N2 and H5N1 viruses, based on assumptions of the mutational rate for influenza (see Materials and Methods). The calculation is based on the total number of virions produced and not the number of infectious virions produced during a typical infection. Thus, whether a virion carrying a drug resistant phenotype will be viable, infectious, and fit enough to grow is not considered. When considering 2-base change variants, the probability of generating all possible combinations was reduced to 22% for H3N2 (Table 1) but did not change for H5N1 (100%). In contrast, when all possible 3-base changes were considered, the probability was reduced by more than a million-fold for both H3N2 and for H5N1, so that the probability of generating a particular triple mutant that could engender resistance to all three drugs used in a combination regime was 1.4×10^−7^ for an H3N2 infection and about 7.2×10^−7^ for an H5N1 infection. The exact values of these probabilities depends on our simplifying assumptions but the general conclusion that generating a particular triple mutant that would engender drug resistance to three drugs is an extremely rare event is a robust conclusion given the influenza mutation rate (see Materials and Methods). These values for influenza A are similar to the probability of generating all possible 1-, 2-, and 3-base changes during the course of a day during chronic HIV infection. Thus, as in HIV, the likelihood of generating drug resistant influenza A variants to a 3-drug regimen is dramatically reduced compared to 1- and 2-drug regimens.

**Table 1:** Probability of Generating all Possible 1-, 2-, or 3-Base Mutations in HIV or Influenza A.

Number of Base Changes	HIV^1^	H3N2^2^	H5N1^2^
1	100%	100%	100%
2	0.7%	22%	100%
3	0.000007%	0.000014%	0.000072%

The rate at which variants of HIV and influenza A viruses are generated which contain 1-, 2-, or 3-base substitutions were calculated using a binomial distribution. Assumptions used for the calculations are provided in Materials and Methods.

1 Per day in chronically infected patients as calculated in [23].

2 Per course of infection in otherwise healthy patients.

### Selection of resistant virus variants under serial passage at fixed concentrations

We then evaluated the emergence of resistant virus upon serial passage in MDCK cells in the presence of fixed concentrations of 1-, 2-, and 3-drug regimens (AMT alone, OSC alone, AMT/OSC double combination, and the TCAD regimen). Drug concentrations were chosen to bracket the clinically relevant concentrations for all three drugs (Table 2 and Materials and Methods). We selected MDCK cells for this experiment for two reasons: i) it is not possible to control the expression of α-2,6-sialyltransferase in MDCK-SIAT1 cells from lot-to-lot, and any variability in expression may affect the levels of resistance to OSC; and ii) the model we are testing is not sensitive to the particular resistance mutant, so that the advantage of the MDCK-SIAT1 over MDCK for producing clinically relevant H274Y variants in neuraminidase does not come into play. We first measured the percentage of variants with AMT resistance-associated substitutions at the end of each passage by quantitative allele-specific PCR (qASPCR). The assays used in this study were specifically designed to detect the V27A, A30T, and S31N substitutions in M2 which confer resistance to amantadine. Due to the large number of conditions tested, data for the multiplicity of infection (MOI) condition resulting in the greatest percentage of resistant virus variants are presented, whereas data for all MOI conditions are reported in Table S1.

**Table 2:** Concentrations (µg/mL) of Drugs Used for Serial Passage at Fixed Concentrations.

Drug	Concentration 11/9× CR	Concentration 21/3× CR	Concentration 3CR*	Concentration 43× CR
Amantadine (AMT)	0.05	0.14	0.43	1.29
Oseltamivir carboxylate (OSC)	0.03	0.10	0.3	0.9
Ribavirin (RBV)	0.14	0.43	1.3	3.9

*CR, clinically relevant (see Materials and Methods).

**Figure 1:**
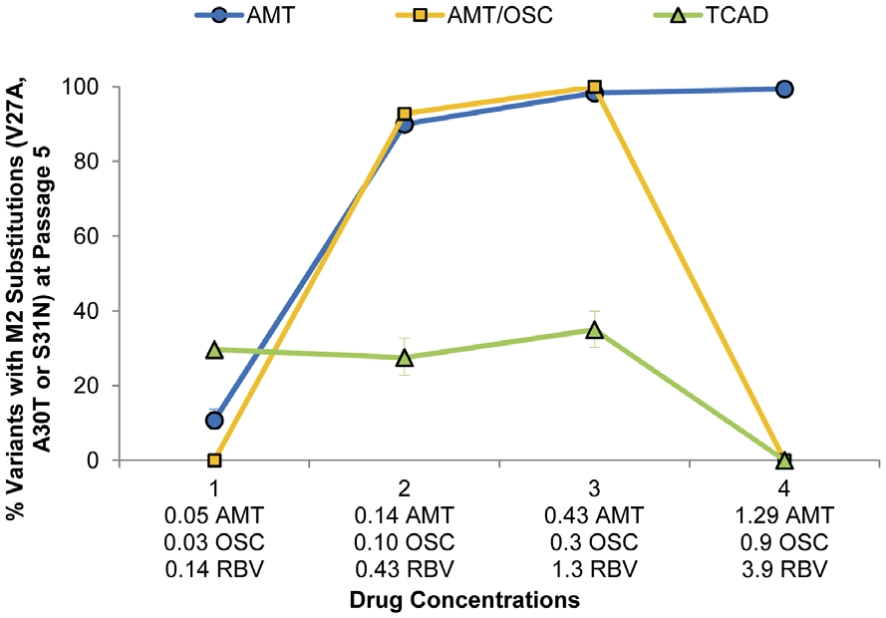
Percent of Resistant Virus Variants Generated at Passage 5 as a Function of Drug Concentration. Wild type influenza A/Hawaii/31/2007 (H1N1) virus was passaged five times in MDCK cells, with the concentrations of drugs in each regimen kept fixed in between passages. The drug concentrations used are given in Table 2 and are discussed in the Materials and Methods. Concentrations 1 to 4 correspond to 1/9, 1/3, 1, and 3 times the clinically relevant concentration for each drug, respectively. The percent of virus variants bearing resistance-associated substitutions in M2 channel (V27A, A30T, or S31N) are presented as the mean of triplicate qASPCR reactions with 95% confidence intervals from a single well of MDCK cells. Concentration 3 represents the clinically relevant concentrations of all three drugs (AMT, OSC, and RBV, see Materials and Methods).

Figure 1 shows the percentage of viruses bearing M2 resistance-associated substitutions at the last passage (passage 5) as a function of drug concentration under the MOI condition resulting in the greatest percentage of resistant virus variants. As shown in Figure 1, treatment with AMT and the AMT/OSC double combination resulted in high percentages of viruses bearing amantadine resistance-associated substitutions in M2 (>98%), with the greatest frequency of resistance occurring at the clinically relevant concentration (Concentration 3) and 1/3 the clinically relevant concentration (Concentration 2). In contrast, treatment with the TCAD regimen resulted in suppression of resistance at all drug concentrations, such that viruses containing M2 resistance-associated substitutions never became predominant in the population (<35%).

Because of the importance of viral load to the absolute number of resistant variants, viral load was also determined for each sample on which qASPCR was performed. Viral load for all samples can be found in Table S1. The no drug control group ranged from 7.9 to 9.1 log_10_ copies/mL at passage 5. Under treatment with AMT alone, the viral load at passage 5 was within 2 log_10_ copies/mL of the matched no drug control in all conditions except for one condition at Concentration 4 (0.01 MOI, 3.1 log_10_ copies/mL reduction). For AMT/OSC at Concentrations 1 and 2, no viral load reduction was seen at any condition at passage 5, regardless of the percentage of resistance-associated substitutions present. At Concentrations 3 and 4, the one condition with high viral load (8.1 log_10_ copies/mL) at passage 5 also had a high percentage of resistance-associated substitutions (100%), whereas conditions which yielded few or no resistant variants (<10%) had viral load reductions of >5.1 log_10_ copies/mL compared to matched no drug control. Treatment with TCAD at Concentrations 1 and 2 in general had little effect on viral load, whereas at Concentrations 3 and 4 there were reductions in viral load of >3.9 log_10_ copies/mL compared to the no drug control. Unlike ATM/OSC, the presence of resistant variants was not correlated with higher viral loads at any concentration. Thus, treatment with TCAD was not only associated with a lower percentage of resistant variants compared to AMT and AMT/OSC, but it also resulted in a decrease in the total number of resistant virions.

**Figure 2:**
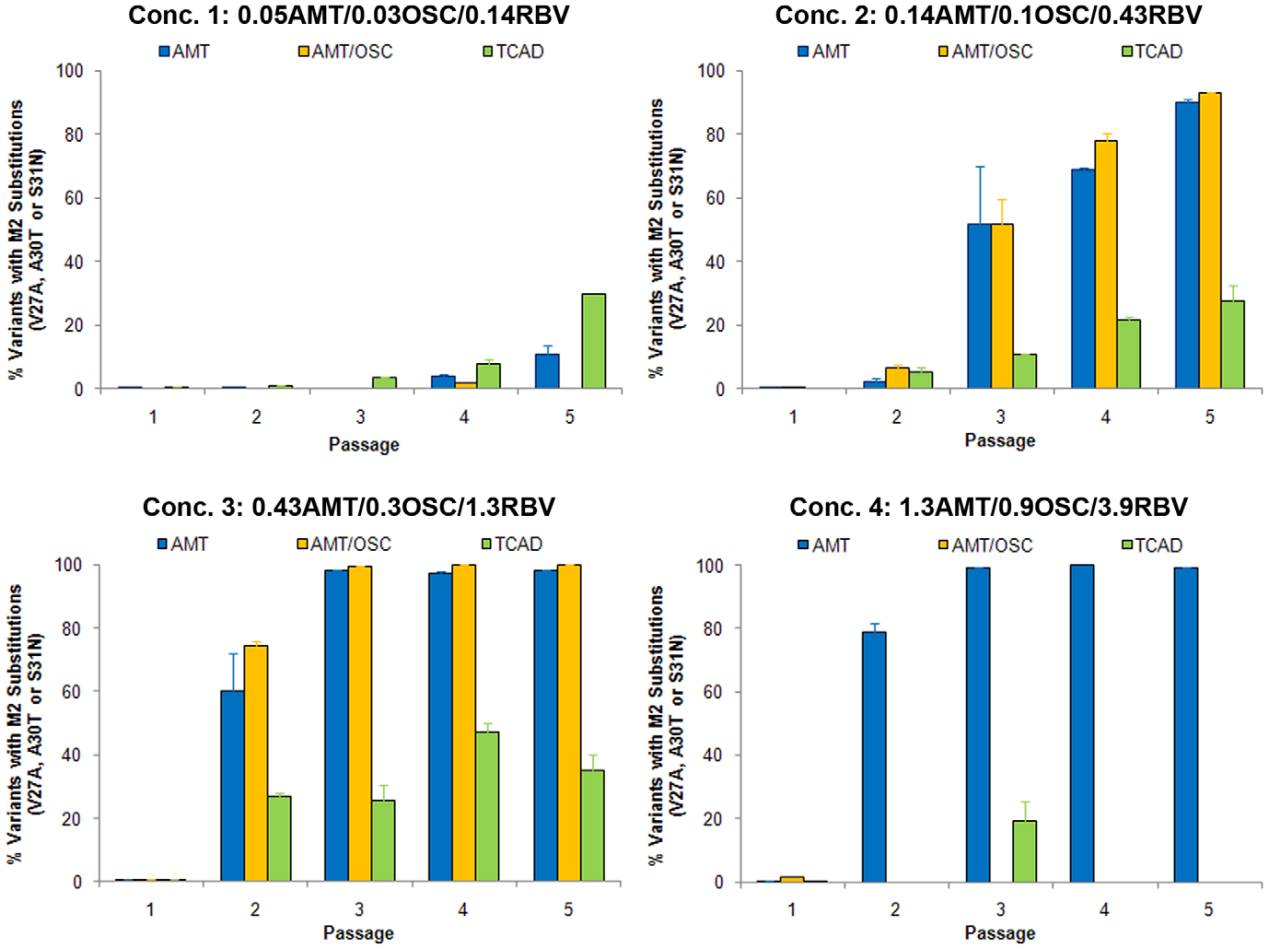
Percent of AMT-Resistant Virus Variants Generated as a Function of Passage Number. Wild type influenza A/Hawaii/31/2007 (H1N1) virus was passaged five times in MDCK cells, with the concentrations of drugs in each regimen kept fixed in between passages. The percent of virus variants bearing resistance-associated substitutions in M2 (V27A, A30T, or S31N) at each passage are presented as the mean of triplicate qASPCR reactions with 95% confidence intervals from a single well of MDCK cells. Each panel represents an increase in the concentration of each drug in the various regimens, with Concentration 3 representing the clinically relevant concentrations of all three drugs (see Materials and Methods). Drug concentrations are provided in units of µg/mL.

Figure 2 shows percentage of virus variants with AMT resistance-associated substitutions as a function of passage at the same four fixed concentrations for the drug regimens which contained AMT (AMT as a single agent, AMT/OSC double combination, and the TCAD regimen). Treatment with AMT alone and the AMT/OSC double combination with Concentration 2 and Concentration 3 (clinically relevant) resulted in the rapid selection of AMT-resistant virus variants, which comprised ≥50% of the population by passage 3 at Concentration 2, and >50% of the population by passage 2 at Concentration 3. Treatment with the TCAD regimen at the same drug concentrations (Concentrations 2 and 3) resulted in sustained suppression of resistance at all passages, such that viruses containing M2 resistance-associated substitutions never became predominant in the population at any time point. The lowest concentrations for all treatment regimens (Concentration 1) resulted in a low frequency and delayed outgrowth of resistant virus variants across all drug regimens at later passages. At the highest concentration (Concentration 4), the AMT/OSC double combination and the TCAD regimen effectively suppressed resistance at all passages, whereas treatment with AMT alone resulted in the predominance of AMT-resistant variants by passage 2.

In order to confirm these results, the serial passage at fixed concentrations was repeated under identical conditions and produced similar results as those presented here, i.e. treatment with AMT and AMT/OSC selected for AMT-resistant variants, whereas treatment with the TCAD regimen resulted in the sustained suppression of resistance (data not shown).

The supernatants from passage 5 of selected conditions were then sequenced by the Sanger method at the M2, hemagglutinin (HA), and NA genes to confirm the results of the qASPCR analysis, and to identify other substitutions which may confer resistance which were not detected by qASPCR. For substitutions in M2, which conferred amantadine-resistance, there was a strong concordance between the genotype as determined by qASPCR and the genotype as determined by Sanger sequence analysis. Of the 14 conditions for which the genotype was determined by both methods, 11 gave consistent results (Table S2). The discrepancies in the other three conditions can be attributed to the specific mutations targeted by the qASPCR assays in this study (only three of the five codons in M2 known to confer resistance were sampled) and/or the differences in the sensitivity for detecting minor populations of the two methods (∼25% for Sanger sequencing and ≤1% for qASPCR). Additional substitutions were detected at position 26 and 34 of the M2 gene upon treatment with TCAD concentration 1 and AMT concentration 4, respectively, which conferred amantadine resistance [26].

Substitutions in HA were also identified by the Sanger method, showing HA substitutions at codons 163 (N163T, H3 numbering) and 165 (S165R) in 9 of 22 and 8 of 22 samples, respectively (Table S2). The N163T substitution was detected in samples treated with regimens containing OSC (OSC alone, AMT/OSC, and TCAD) but not in samples treated with AMT alone, whereas the S165R substitution was detected in samples treated with any regimen (AMT alone, OSC alone, AMT/OSC, and TCAD). Both of the HA substitutions confer high level resistance to OSC and ZAN, but not AMT (see below). The NA gene was also sequenced though no substitutions were detected.

### Selection of resistant virus variants under serial passage at escalating concentrations

**Figure 3:**
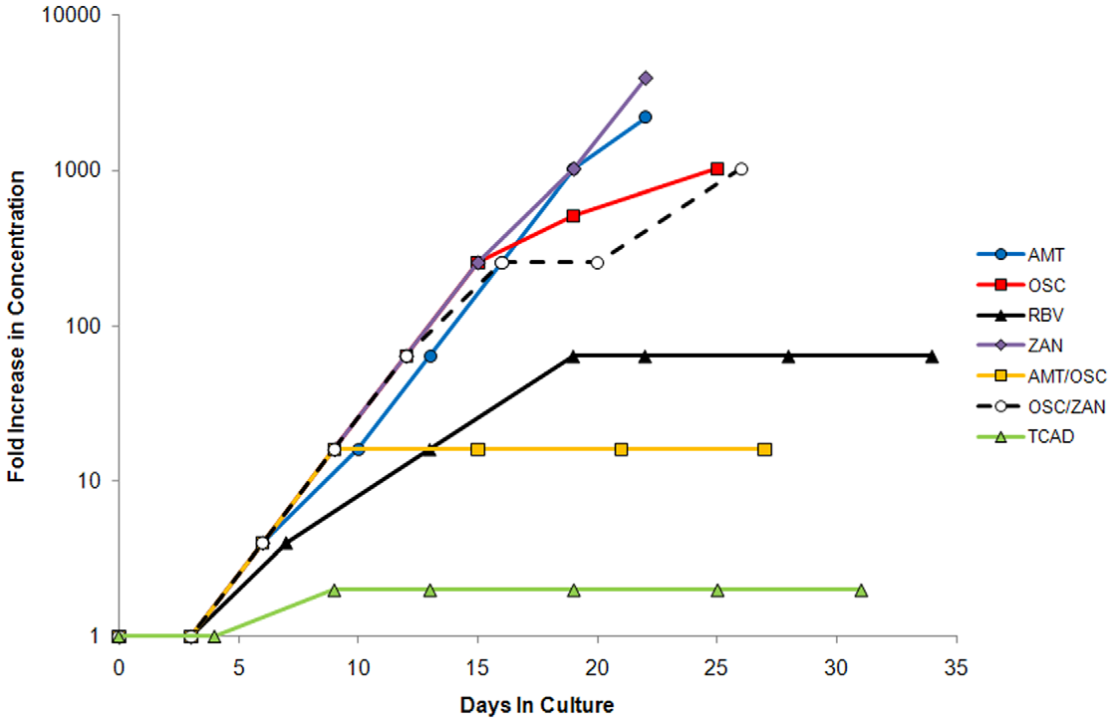
Passage History of Wild Type Influenza A/Hawaii/31/07 under Escalating Drug Concentrations. Wild type influenza A/Hawaii/31/2007 (H1N1) virus was passaged in the presence of escalating concentrations of each drug regimen for a total of ≥25 cumulative days in culture, or until the drug concentration reached the 50% of the cytotoxic concentration (TC_50_) of the drug as a single agent.

The selection for resistant virus variants upon serial passage in MDCK cells under escalating concentrations of drug regimens (single agents and double and triple combinations) was examined by monitoring the susceptibility of the virus to each drug after serial passage. Figure 3 shows the fold increase in concentration of each drug regimen versus cumulative days in culture (see Table S3 in Supporting Material for more details of the passage history). The virus was passaged for a total of 22–31 days in the presence of each drug regimen. For AMT and ZAN, serial passaging was terminated when the drug concentration reached the TC_50_ (33 µg/mL for AMT; for ZAN, since the TC_50_ had not been previously determined, the TC_50_ of 118 µg/mL for OSC was used as the predetermined not-to-exceed concentration).

In the presence of AMT, OSC, and ZAN as single agents, the virus was able to replicate under increasing drug concentrations at every passage starting at passage 2 until the last passage. The final drug concentrations at the last passage were 33 µg/mL for AMT (2200-fold starting concentration), 30.7 µg/mL for OSC (1024-fold starting concentration), and 118 µg/mL for ZAN (3933-fold starting concentration). In the presence of RBV as a single agent, the highest concentration that resulted in virus-induced cytopathic effect (CPE) was 96 µg/mL (64-fold starting concentration), at which point further increases in concentration resulted in the inhibition of CPE. The virus was passaged for a total of 4 passages (15 days total) at 96 µg/mL RBV without further increases in drug concentration.

In the presence of the AMT/OSC double combination, the highest concentration that resulted in virus-induced CPE was 0.24 µg/mL AMT and 0.48 µg/mL OSC (16-fold the starting concentration for each drug), at which point further increases in concentration resulted in the inhibition of CPE. In the presence of the OSC/ZAN double combination, the virus was able to replicate under steadily increasing drug concentrations, such that final drug concentrations at the last passage were 30.7 µg/mL OSC and 30.7 µg/mL ZAN (1024-fold starting concentration of each drug).

In the presence of the TCAD regimen, the highest concentration that resulted in virus-induced CPE was 0.03 µg/mL AMT, 0.06 µg/mL OSC, and 3.0 µg/mL RBV (2-fold starting concentration for each drug), at which point further increases in concentration resulted in the inhibition of CPE. The virus was passaged for a total of 5 passages (22 days total) at this concentration without further increases in drug concentration.

### Phenotypic resistance under serial passage at escalating drug concentrations

The phenotypic resistance of the viruses in the supernatants after serial passage in the presence of increasing drug concentrations was determined by monitoring the susceptibility to individual drugs, and resistance is defined as a shift in the 50% effective concentration (EC_50_) of >5-fold compared to the input virus. Table 3 provides a summary of the measured EC_50_ value and fold-change in EC_50_ for each drug regimen. Serial passage of the virus in the absence of drugs (no drug control) resulted in increases of 2.2-fold, 34-fold, 1.4-fold, and 3.3-fold in EC_50_ to AMT, OSC, ZAN, and RBV, respectively, relative to the input virus. These data demonstrate that the virus remained susceptible to AMT and RBV. The EC_50_ values of OSC and ZAN varied widely (>40-fold) between replicate plates (data not shown), and thus no conclusions regarding the susceptibility of the viruses in the supernatant to these drugs could be made. However, when individual clones were plaque purified from the no drug control and tested, the EC_50_ values for OSC and ZAN were within 0.3- to 0.9-fold of the input virus (Table 4), indicative that these viruses were sensitive to OSC and ZAN.

**Table 3:** Summary of Phenotype and Genotype of Supernatants after Serial Passage under Escalating Drug Concentrations.

Regimen	EC_50_ (µg/mL)^a^	Fold Change in EC_50_ vs Input Virus	Substitution Detected^b^ (Protein)
No Drug Control	AMT = 0.089±0.076	2.2	D190N (HA)
	OSC = 1.2±1.6	34	
	ZAN = 1.1±1.5	1.4	
	RBV = 5.3±1.6	3.3	
AMT	AMT = >33	>800	L26F (M2)
OSC	OSC = 13±11	371	S165R (HA)
ZAN	ZAN = 33±28	43	S165R (HA)
RBV	RBV = 3.6±2.4	2.2	–
AMT/OSC	AMT = 0.072±0.039	1.8	S165R (HA)
	OSC = 1.6±1.8	46	
OSC/ZAN	OSC = 10±7.4	286	S165R (HA)
	ZAN = 17±12	22	
TCAD	AMT = 0.78±0.027	2.0	N163T (HA)
	OSC = 48±34	1371	
	RBV = 5.5±1.5	3.4	

Wild type influenza A/Hawaii/31/07 (H1N1) virus was passaged in the presence of escalating concentrations of each drug regimen for a total of ≥25 cumulative days in culture, or until the drug concentration reached the 50% of the cytotoxic concentration (TC_50_) of the drug as a single agent. Each drug regimen was tested starting at 1/8^th^ the EC_50_ of each drug as a single agent. The drug concentration was increased 1X, 2X, or 4X at each passage starting at passage 2, depending on whether virus-induced cytopathic effect (CPE) was apparent during the passage. Phenotypic testing using neutral red assay in MDCK cells was performed on the supernatants from each regimen to determine the susceptibility to each drug as a single agent after serial passage. In addition, the supernatant from selected passages from each regimen was sequenced by the Sanger method at the M2, hemagglutinin (HA), and/or neuraminidase (NA) genes to detect the presence of mutations.

a EC_50_ values are the mean of 6 replicate wells from two plates with standard deviations.

b Amino acid positions in HA are designated using universal H3 numbering [42].

**Table 4:** Susceptibility of Virus Clones to AMT, OSC, RBV and ZAN as Single Agents.

	AMT		OSC		RBV		ZAN	
Substitution (Regimen)	EC_50_ (µg/mL)^a^	Fold increase relative to input virus	EC_50_ (µg/mL)^a^	Fold increase relative to input virus	EC_50_ (µg/mL)^a^	Fold increase relative to input virus	EC_50_ (µg/mL)^a^	Fold increase relative to input virus
Input virus	0.040±0.013	-	0.035±0.014	-	1.6±0.18	-	0.77±0.47	-
Wild type (NDC)^b^	0.055±0.004	1.4	0.030±0.006	0.9	1.2±0.13	0.8	0.24±0.064	0.3
M2-L26F (AMT)	>33	>800	0.005±0.001	0.14	0.65±0.064	0.4	0.078±0.046	0.10
HA-S165R (OSC)^c^	0.078±0.059	2.0	11±2.8	330	2.1±0.22	1.3	49±21	64
HA-N163T (TCAD)^c^	0.029±0.001	0.7	20±1.9	560	1.1±0.16	0.7	104±28	140

Wild type influenza A/Hawaii/31/07 (H1N1) virus was passaged in the presence of escalating concentrations of each drug regimen, and the supernatant from selected passages from each regimen was sequenced by the Sanger method at the M2, hemagglutinin (HA), and/or neuraminidase (NA) genes to detect the presence of mutations. Six virus clones were isolated from each regimen (from the latest passage that yielded viable plaques) and were sequenced at M2, HA, and NA to confirm that the genotype of the clone matched that of the supernatant. The susceptibility of clones bearing representative substitutions to AMT, OSC, RBV, and ZAN was tested using neutral red assay.

a Each drug was tested using two plates, with 3 replicate wells per plate. EC_50_ values are the mean of duplicate plates with standard deviations.

b No drug control passaged in parallel.

c Amino acid positions in HA are designated using universal H3 numbering [42].

Serial passage in the presence of AMT, OSC, and ZAN as single agents resulted in resistance to each of these drugs, as demonstrated by the increases of >43-fold in EC_50_ values to each drug relative to the input virus. In contrast, serial passage in the presence of RBV as a single agent resulted in an EC_50_ shift of 2.2-fold relative to the input virus. In the presence the AMT/OSC double combination, serial passage resulted in a 1.8-fold change in EC_50_ for AMT and a 46-fold change in EC_50_ for OSC, indicative of resistance to OSC but not AMT. Serial passage in the presence of OSC/ZAN double combination resulted in a >22-fold change in EC_50_ values to both drugs, indicative that resistance emerged to both drugs. For the TCAD regimen, serial passage resulted in a 2-fold, 1371-fold, and 3.4-fold increase in EC_50_ values to AMT, OSC, and RBV, respectively. These data indicate resistance was generated to OSC but not to AMT or RBV.

### Effects of substitutions on drug susceptibility

The presence of mutations in the M2, HA, and or NA genes of viruses in the supernatant after serial passage in the presence of escalating drug concentrations were detected by Sanger sequencing. A summary of amino acid substitutions detected in supernatants is provided in Table 3. Genotypic analysis of the supernatant from the no drug control identified the D190N amino acid substitution in HA. Although an asparagine at position 190 in HA marks a change in sequence from the input virus, the presence of asparagine at position 190 is consistent with the published sequence for A/Hawaii/31/07 (H1N1). Phenotypic analysis of the virus clones purified from the no drug control revealed that the EC_50_ values for AMT, OSC, RBV, and ZAN were within 0.3- to 1.4-fold of the input virus, suggestive that the D190N substitution does not confer reduced susceptibility. No substitutions were identified in M2 or NA for the no drug control.

Genotypic analysis of supernatants passaged in the presence of AMT, OSC, and ZAN as single agents identified single amino acid substitutions – L26F in M2 in the case of AMT, and S165R in HA in the case of OSC and ZAN. Phenotypic analysis using purified virus clones confirmed that these substitutions conferred high level resistance to these drug regimens (Table 4). The purified virus clone bearing the L26F substitution showed an increase of >800-fold in EC_50_ value for AMT as compared to the input virus. Similarly, the purified virus clone bearing the S165R substitution showed an increase of 330-fold in EC_50_ value for OSC and 64-fold in EC_50_ value for ZAN as compared to the input virus. In contrast, no amino acid substitutions were identified in the supernatants passaged in the presence of RBV (for M2, HA, or NA), and virus clones isolated from the RBV regimen remained susceptible to RBV (data not shown).

Serial passage in the presence of escalating concentrations of AMT/OSC and OSC/ZAN resulted in the selection of viruses bearing a single amino acid substitution (S165R in HA) which conferred high level resistance to OSC and ZAN but not to AMT (Tables 3 and 4). For the AMT/OSC regimen, a second substitution in HA (Y178F) was identified at passage 5 after 21 days in culture (Table S3). However, it was not possible to isolate virus clones bearing the Y178F substitution, and thus the effect of this substitution on drug susceptibility is unknown.

In the presence of escalating concentrations of the TCAD regimen, serial passage resulted in the emergence of viruses bearing the N163T substitution in HA. The N163T substitution conferred resistance to OSC and ZAN, but not to AMT or RBV. The EC_50_ value of OSC and ZAN against the purified virus clone bearing the N163T substitution was 560- and 140-fold higher, respectively, than that of the input virus. In contrast, the EC_50_ values for AMT and RBV against the purified N163T variant were within 0.7-fold of the EC_50_ values of the input virus (Table 4).

### Contribution of each drug in TCAD to the suppression of resistance

The contribution of each of the three drugs in the TCAD regimen – AMT, OSC, and RBV – to the suppression of resistance was examined by quantifying the frequency of virus breakthrough under selective pressure from each double combination and the TCAD regimen using fixed clinically relevant concentrations of two drugs and varying concentrations of each drug as the third drug.

**Figure 4:**
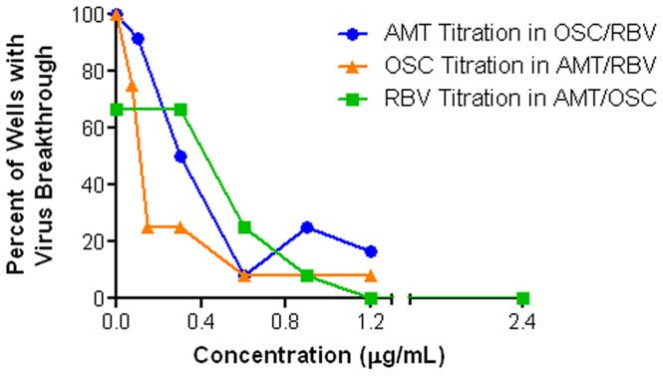
Each Drug in TCAD Contributes to the Suppression of Virus Breakthrough. MDCK cells in 96-well plates were infected with influenza A/Hawaii/31/2007 (H1N1) virus in the presence of a combination of two drugs at fixed concentrations with varying concentrations of the third drug, using 12 replicates for each condition. The fixed concentrations of the double combinations were 0.30 µg/mL OSC and 0.60 µg/mL RBV, 0.6 µg/mL AMT and 0.6 µg/mL RBV, or 0.6 µg/mL AMT and 0.3 mg/mL OSC. Following 5 serial passages, the number of wells for each condition having virus breakthrough, defined as >50% cytopathic effect, was determined by neutral red uptake.

Figure 4 shows the percentage of wells exhibiting virus breakthrough as a function of increasing concentrations of each titrated third drug. The results show that treatment with all three double combinations – OSC/RBV, AMT/RBV, and AMT/OSC – resulted in a high percentage of wells having virus breakthrough (75–100%). Titration of each drug into the appropriate double combination resulted in the concentration-dependent decrease in the number of wells with virus breakthrough. A statistical analysis of the number of wells with virus breakthrough demonstrated that each drug made a statistically significant contribution to the suppression of virus breakthrough at clinically achievable concentrations: AMT at 0.3 µg/mL and above, OSC at 0.15 µg/mL and above, and RBV at 0.9 µg/mL and above (Table 5). When the supernatants from wells with virus breakthrough were sequenced by the Sanger method, there was a high concordance between virus breakthrough and the presence of resistance-associated substitutions in M2, HA, and /or NA (Table 5). Specifically, all the substitutions detected occurred at positions that have been demonstrated to confer resistance to amantadine (amino acid positions 26, 27, 30, 31, and 34 in M2) and/or oseltamivir (274 in NA, and 163 and 165 in HA).

**Table 5:** Virus Breakthrough and the Presence of Resistance-Associated Substitutions as a Function of the Concentration of the Third Drug in TCAD.

Concentration of Third Drug (µg/mL)	No. Wells with Virus Breakthrough	P value^a^	No, Wells with Substitutions^b^	Amino Acid Substitutions^c^
Titration of amantadine into fixed concentrations of OSC/RBV				
0	12	–	11	M2: L26F; HA: N163T, S165R
0.1	11	0.761	11	M2: V27A, A30T, S31N, G34E;NA: H274Y; HA: N163T, S165R
0.3	6	0.034	5	M2: V27A, A30T, S31N; NA: H274Y
0.6	1	<0.001	1	M2: G34E; HA: N163T
0.9	3	0.001	3	M2: S31N; HA: N163T, S165R
1.2	2	<0.001	2	M2: S31N; HA: N163T
Titration of oseltamivir carboxylate into fixed concentrations of AMT/RBV				
0	12	–	11	M2: V27A, A30T, S31N, G34E
0.075	9	0.109	8	M2: L26F, V27G, G34E;HA: N163T, S165R
0.15	3	<0.001	3	M2: S31N; HA: S165R
0.3	3	<0.001	3	M2: L26F, V27A, G34E; HA: N163T
0.6	1	<0.001	1	M2: S31N
1.2	1	<0.001	1	M2: S31N
Titration of ribavirin into fixed concentrations of AMT/OSC				
0	8	–	7	M2: V27G; V27A, S31N; HA: N163T
0.3	8	0.6667	8	M2: V27A, A30V; HA: N163T, S165R
0.6	3	0.0498	0	
0.9	1	0.0047	1	M2: S31N
1.2	0	0.0007	0	
2.4	0	0.0007	0	

MDCK cells in 96-well plates were infected with influenza A/Hawaii/31/2007 (H1N1) virus in the presence of a combination of two drugs at fixed concentrations with varying concentrations of the third drug, using 12 replicates for each condition. The fixed concentrations of the double combinations were 0.30 µg/mL OSC and 0.60 µg/mL RBV, 0.6 µg/mL AMT and 0.6 µg/mL RBV, or 0.6 µg/mL AMT and 0.3 µg/mL OSC. Following 5 serial passages, the number of wells for each condition having virus breakthrough, defined as >50% cytopathic effect (CPE), was determined by neutral red uptake.

a Statistical analysis of the number of wells with virus breakthrough at each concentration of third drug compared to no third drug was performed using the Fisher's exact test.

b The supernatants from wells with virus breakthrough were analyzed by Sanger sequencing at the M2, HA, and NA genes to determine the presence of resistance-associated mutations.

c Subtitutions are listed if they were detected in any well by Sanger sequencing. Amino acid positions for NA and HA are presented using N2 and H3 numbering, respectively.

## Discussion

In this report, we present a model system and process to evaluate the effects of drug combinations on the emergence of resistance. While the experimental conditions utilized may not reflect conditions *in vivo*, they provide a basis to compare the effects of different drug regimens under similar conditions. The use of mathematical models, multiple methods to generate resistance, and multiple methods to quantitate the effects of antiviral drug regimens on resistance enabled an in depth analysis of the effects of single and combination drug therapies on the virus population. The process and experimental designs described herein may provide a generalizable strategy for the optimization of drug combinations and concentrations to suppress resistance in other viral diseases. The results from all the methods employed consistently demonstrated that a combination of three drugs was superior to single or dual agents at suppressing resistance in influenza A viruses.

There is currently little data on the effects of antiviral combinations on the emergence of resistance in influenza. Ilyushina et al. [27] tested the effects of the double combination of OSC and AMT on the development of resistance in three influenza A subtypes (H1N1, H3N2, and H5N1) *in vitro*. In that study, serial passage at increasing concentrations of AMT or OSC alone resulted in high virus yield at the 5^th^ passage, and the viruses from the supernatant of the last passage bore substitutions in M2 (AMT-treated) and HA (OSC-treated). In contrast, treatment with the AMT/OSC double combination resulted in low or undetectable viral titer, and no substitutions in either M2 or HA were identified at the higher concentrations of both drugs. One caveat is that the study utilized supra-physiological concentrations of AMT. The lowest concentration of AMT used was 10 µM (1.9 µg/mL), which is 3- to 4-fold higher than what could be achieved in plasma based on labeled doses (see Materials and Methods), and thus the results may not be clinically meaningful. In a clinical study, Ison et al. [28] reported the use of nebulized ZAN in combination with rimantadine in adults hospitalized with influenza infection. Although the study was terminated early and was underpowered, the authors noted that 2 of the 21 patients receiving rimantadine monotherapy developed rimantadine resistant virus variants with A30T and S31N substitutions in M2, whereas 0 of the 20 patients treated with rimantadine in combination with ZAN developed resistance to either drug.

To first determine the number of drugs required in a regimen to suppress the emergence of resistance in influenza A virus, we calculated the probability of an infecting drug-sensitive virus acquiring all the necessary mutations to escape the effects of 1, 2, or 3 drugs. The results predict that a three-drug regimen would have significant therapeutic benefit over one- and two-drug regimens as some or all of the drugs are likely to remain active during the course of treatment. However, if a fit 2-base change variant was generated early in infection, it could further acquire an additional mutation. Our calculation ignores this possibility and thus the dangers of drug resistance emergence on therapy could be greater than estimated (Table 1). While these results need to be confirmed in clinical trials for influenza, the benefit of a three-drug regimen over one- and two-drug regimens have been demonstrated in clinical trials in HIV [29], [30], [31] as well as in clinical practice [32].

We then quantified the levels of resistance to AMT after serial passage under pressure from fixed concentrations of drugs, using qASPCR to detect and quantitate markers of resistance. As resistance to AMT is not complicated by assay discrepancies, i.e. substitutions in M2 confer resistance to AMT both in cell culture assays and *in vivo*, the use of qASPCR to quantitate AMT resistance may provide a better representation of the total resistance under each treatment regimen. As expected, resistance to AMT as a single agent emerged rapidly. Surprisingly, the AMT/OSC double combination was not superior to AMT alone at suppressing resistance to AMT; treatment with AMT/OSC paralleled treatment with AMT alone in the rate in which resistance emerged and the percentage of resistant variants in the population (Figure 2). In contrast, the TCAD regimen was effective at suppressing resistance to AMT at all concentrations tested. It should be noted that Abed et al. have shown that variants bearing substitutions in M2 do not have reduced fitness [33]. At Concentration 3 where maximum resistance was observed in the AMT and AMT/OSC conditions, variants with high level resistance to AMT also emerged under treatment with the TCAD regimen but did not out-compete the wild type population, suggesting that a single substitution in M2 did not confer a selective advantage. Furthermore, the observation that there was a stronger selective pressure for AMT-resistant variants under treatment with the AMT/OSC double combination than the TCAD regimen clearly shows that the addition of RBV to AMT/OSC double combination had a profound impact on the suppression of AMT resistance. We have previously shown that AMT contributed to the antiviral activity of the TCAD regimen against AMT-resistant influenza A viruses at clinically achievable concentrations where AMT had no activity as a single agent [21]. The lack of a strong selective advantage for variants with M2 substitutions under TCAD treatment may be partially due to the enhanced activity of AMT as part of the TCAD regimen against the AMT-resistant variants.

Sanger sequence analysis of the supernatants from the last passage of the fixed combination study revealed the presence of substitutions in M2 and HA. The generation of variants with HA substitutions as the result of serial passage in the presence of NAIs have been documented previously [11], [12], [27], and resistance to NAIs *in vitro* may arise as the result of a lower affinity for cellular receptors with a concomitant decrease in dependence on NA for release from the cell surface [11], [34]. It should be noted that while HA substitutions may confer resistance to NAIs in cell culture assays, these substitutions do not necessarily confer reduced susceptibility when evaluated using neuraminidase enzyme inhibition assays or in *in vivo* models [35]. While substitutions in HA which confer resistance to NAIs may be a phenomenon specific to cell culture assays, they are relevant in this context as they represent a pathway to resistance which can be used to evaluate the effects of different drug regimens.

In this study we demonstrated that, under serial passage at escalating concentrations, the TCAD regimen imposed a high genetic barrier to resistance. This is demonstrated by the fact the single substitution (N163T in HA) that emerged under TCAD treatment conferred resistance to OSC, but not to AMT and RBV, and thus was not adequate to allow the virus variants to escape the effects of the other drugs in the TCAD regimen (Tables 3 and 4). The AMT/OSC regimen also imposed a higher barrier to resistance compared to the single agents and double NAI combination (OSC/ZAN), in which a single substitution conferred resistance to OSC but not to AMT. We did not evaluate the genetic barrier to resistance for RBV in this study, as phenotypic resistance to RBV was not detected (Table 3). While the virus was able to replicate at up to 96 µg/mL RBV in the serial passage experiment, which is 10- to 20-fold higher than the EC_50_ of RBV, it should be noted that each passage had a duration of up to 6 days in culture, whereas the incubation for EC_50_ determination was 3 days. Thus, it is possible that replication by susceptible viruses is capable of causing CPE at higher RBV concentrations after 6 days, which would not be detected by EC_50_ determination after 3 days. The lack of resistance to RBV is not surprising, given that resistance to RBV is rare in other viruses and clinical resistance to RBV in HCV has not been clearly demonstrated [36], [37]. To our knowledge, resistance to RBV in influenza viruses has not been identified.

Finally, we also examined the contribution of each drug in the TCAD regimen to the suppression of virus breakthrough and resistance using 12 replicate wells per condition, which allowed us to measure the frequency of virus breakthrough. Statistically significant reductions in virus breakthrough were observed in all cases where each drug was titrated as the third drug into the double combinations under clinically achievable concentrations. Thus, each drug in the TCAD regimen made a concentration-dependent contribution to the suppression of resistance. Importantly, these data enable the determination of the optimal concentrations of each drug in the TCAD regimen for maximal suppression of resistance.

These data also help establish a paradigm for an integrated process coupling mathematical modeling with multiple experimental designs to better understand the relationship between viral dynamics and evolution of resistance under combination drug pressure. Future work should attempt to i) confirm these findings using *in vivo* models, ii) explore the interplay between the synergy of drug combinations and barriers to resistance in wild type and previously resistant viruses, iii) and extend these findings to other rapidly mutating pathogens for which combination approaches are desperately needed.

## Materials and Methods

### Modeling mutation in HIV and influenza virus

Starting from a drug-sensitive or wild-type virus the number of HIV and influenza A viruses that contain 1-, 2-, or 3-base substitutions were calculated using a binomial distribution where we assumed: 1) the mutation rate for influenza A virus is 2×10^−6^ per nucleotide per infectious cycle [38]; and 2) the number of bases in the influenza A genome is 1.4×10^4^. Much higher mutation rates have also been reported [24], [25] and thus we are being conservative in estimating the error rate involved in RNA replication. We further assumed that 3) there are 4×10^8^ epithelial cells in the upper respiratory tract [39], 25% of which become infected before influenza is cleared [40]; 4) each infected cell produces ∼5000 virions, so that the total number of virions produced during infection is ∼5×10^11^ (higher levels of viral production per cell have been reported by Mohler et al. [41] so this estimate may be conservative); and 5) for H5N1 viruses, it is assumed that the infection can spread to the lower respiratory tract and infect 5-fold more cells. Comparing the number of virions produced carrying 1, 2, or 3 mutations with the total number of possible variants yields the entries in Table 1. The calculation for HIV is taken from [23].

### Antiviral compounds

Amantadine (AMT) was obtained from Moehs Catalana, S.I. (Barcelona, Spain). Oseltamivir carboxylate (OSC, the active metabolite of oseltamivir) was obtained from Charnwood Molecular (Loughborough, United Kingdom) through synthesis via the Nboc-protected acid from oseltamivir phosphate. Zanamivir (ZAN) was obtained from Haorui Pharma-Chem, Inc. (Edison, NJ). Ribavirin (RBV) was purchased from BASF Orgamol Pharma Solutions SA (Evionnaz, Switzerland).

### Influenza virus preparation

The virus used for this study was wild type influenza type A/Hawaii/31/2007 (H1N1) and was obtained from the Centers for Disease Control and Prevention (Atlanta, GA). The CDC stock was sequentially passaged two times in MDCK cells to produce a single virus stock. This virus stock was then aliquoted and stored at −80°C and used for antiviral studies. Sanger sequencing of the M2, HA, and NA genes from the virus stock revealed identity of 100% in the M2 protein, 99% in the HA protein, 100% NA proteins with published sequences (reference EU516164.1 for M2, EU516078.1 for HA, and EU516128.1 for NA). The HA protein had a single amino acid change (N190D, H3 numbering [42]) compared to the published sequence.

### Cell preparation

MDCK cells (Madin Darby canine kidney cells, ATCC cat. no. CCL-34) were grown in 150 cm^2^ flasks in MEM/EBSS medium (Hyclone Laboratories, Logan, UT) and 5% heat inactivated fetal bovine serum (Hyclone Laboratories) from a frozen aliquot of a working cell bank originating from the ATCC stock and the lineage replaced every two months. Within this time frame the number of cell passages is no more than 10 passages beyond the ATCC stock.

On the day preceding the experiment, the cells were removed from the 150 cm^2^ flask by trypsinization using 0.25% trypsin with 0.2 g/L EDTA (Hyclone Laboratories), and measured for count and viability by hemacytometer reading in 0.4% trypan blue (Sigma-Aldrich, St. Louis, MO). MDCK cells were resuspended to 4×10^5^ cells per mL in tissue culture medium (specified above) and added to 6-well or 96-well plates as appropriate. The plates were covered with a plate cover and incubated at 37°C and 5% CO_2_ overnight to allow for cell adherence. Prior to infection, the monolayers were washed with MEM/EBSS to remove residual fetal bovine serum.

### Serial passage at fixed concentrations of drug regimens

Each well of the 6-well plates contained 4.0 mL of test solution [50 µg/mL gentamicin (Sigma-Aldrich), 1 µg/mL EDTA (Sigma-Aldrich), and 10 U/mL trypsin (Sigma-Aldrich) in MEM/EBSS medium] with the appropriate virus dilution with or without drug(s). Briefly, the cells were incubated in the presence of the test solution for 15 minutes at 37°C and 5% CO_2_, and were washed twice with 2 mL of pre-warmed medium containing the appropriate drug regimen to remove unbound viruses, and then replenished with 4 mL pre-warmed medium containing drug and incubated for 3 days at 37°C and 5% CO_2_. After day 3, the cells were scraped off the well and collected along with the supernatant, briefly sonicated in 30 second bursts to release cell associated virus, and then aliquoted and stored at −80°C for subsequent serial passage. Each condition (drug regimen, concentration, and MOI) was tested in a single well of a 6-well plate.

Virus was passaged using three different MOIs (0.1, 0.01, and 0.001) in the presence of fixed concentrations of each drug regimen for a total of 5 passages, with each passage having a duration of 3 days. After each passage, the virus titer was determined for each condition (drug regimen, concentration and MOI) by end point titration as previously described [22] and the virus was diluted to achieve targeted MOIs for the next passage. If the titer was too low to achieve the targeted MOI, 0.4 mL of the undiluted cell preparation from the preceding passage was used to infect cells at the next passage. Subsequent infections were performed as described above.

The drug regimens tested included AMT and OSC as single agents, AMT/OSC in double combination, and the TCAD regimen. Each drug regimen was tested at four concentrations that bracketed the predicted average human plasma concentrations (C_ave_) of each drug, with double and triple combination regimens tested as fixed ratios of drugs that are the same concentrations as the single agent regimens. Based on pharmacokinetic data in humans (manuscript in preparation) and simulation (data not shown), the labeled dose of 100 mg twice a day for amantadine, 75 mg twice a day for oseltamivir phosphate, and 600 mg twice a day for ribavirin is expected to produce a C_ave_ of 0.43 µg/mL for AMT, 0.3 µg/mL for OSC (oseltamivir carboxylate, the active metabolite), and 1.3 µg/mL for RBV in the first 10 days. Concentrations of drug used for serial passage at fixed concentrations are provided in Table 2.

The percentage of resistance-associated mutations in a sample was determined by allele-specific real-time PCR (ASPCR) using TaqMan® assays that detected the V27A, A30T, and S31N substitutions in M2 for amantadine resistance. ASPCR was performed at each passage and for each condition. Sanger sequence analysis of the M2, NA, and HA genes were performed on a subset of samples to confirm the ASPCR results and to determine whether additional mutations occurred at codons not monitored by the ASPCR (see below). For experiments with serial passage at fixed drug concentrations, Sanger sequence analysis was performed on supernatants from passage 5.

### Serial passage at escalating concentrations of drug regimens

MDCK cells were seeded into 6-well plates in a volume of 3 mL and the monolayers were washed with MEM/EBSS to remove residual fetal bovine serum as described above. The virus stock (influenza A/Hawaii/31/2007 (H1N1)) was added to obtain a final in-well initial MOI of 33×10^−6^ CCID_50_/cell. The assay plates were covered with a plate cover and incubated for 15 minutes at 37°C and 5% CO_2_ to allow virus adsorption. The supernatant was then removed and the cells were washed twice with 2 mL of pre-warmed (37°C) assay medium containing the appropriate drug regimen and replenished with 4 mL of assay medium containing the appropriate drug regimen.

The drug regimens tested include AMT, OSC, ZAN, and RBV as single agents; the double combinations of AMT/OSC and OSC/ZAN; and the TCAD regimen. The starting concentrations for each drug regimen was 1/8^th^ the EC_50_ of each drug as a single agent as determined previously (data not shown). Single agents were tested at starting concentrations of 0.015 µg/mL AMT, 0.03 µg/mL OSC, 0.03 µg/mL ZAN, or 1.5 µg/mL RBV; the AMT/OSC double combination at 0.015 µg/mL AMT plus 0.03 µg/mL OSC; the OSC/ZAN double combination at 0.03 µg/mL OSC plus 0.03 µg/mL ZAN; and the TCAD regimen at 0.015 µg/mL AMT, 0.03 µg/mL OSC, and 1.5 µg/mL RBV. The virus was passaged once at the starting concentration for each drug regimen. At the second passage, the supernatant was diluted 1∶1000 in MEM/EBSS and 100 µL of the diluted supernatant from each regimen was used to infect 3 wells containing drug concentrations that were 1-fold, 2-fold, and 4-fold the drug concentration used in the first passage. At the end of the second passage, the supernatant from the highest concentration of drug that achieved ≥50% cytopathic effect (CPE, measured using neutral red uptake as described below) was used to infect three wells containing drug concentrations that were 1-fold, 2-fold, and 4-fold the highest concentration that achieved ≥50% CPE in passage 2. Each passage had a duration of ≤6 days: the cell monolayers were examined everyday for the presence of CPE, and on the day in which ≥50% CPE was observed for a well, the supernatant was collected and stored at −80°C. This procedure was repeated for all subsequent passages. Passaging was continued until the drug concentrations reached the 50% cytotoxic concentration (TC_50_) for each drug as a single agent (33 µg/mL for AMT, 118 µg/mL for OSC, and 677 µg/mL for RBV) or the virus had spent ≥25 days cumulatively in culture in the presence of drugs, whichever came first.

The susceptibility to AMT, OSC, ZAN, and RBV, was performed on the supernatants from each regimen (see below). Drug susceptibility testing was performed using the supernatants from the latest passage that yielded a sufficiently high virus titer to allow dilution of the drug in the supernatant to a concentration that did not interfere with the susceptibility assay (≥1000-fold dilution). In addition, phenotypic analyses were performed on selected clones bearing representative amino acid substitutions identified from Sanger sequencing to determine the effects of the substitution on drug susceptibility.

Sanger sequence analysis of the M2, NA and HA genes were performed on supernatants from passage 3 and the last passage for each drug regimen. If a mutation was detected, the supernatants from additional passages were analyzed by the Sanger method to identify the first appearance of the mutation. For each regimen, six virus clones were obtained by plaque purification and were sequenced to confirm that the genotype matched the genotype of the supernatant. The clones were obtained from the latest passage that yielded plaques. Drug susceptibility testing was performed on selected clones to determine the effects of the mutation on drug susceptibility.

### Contribution of each drug in TCAD to the suppression of viral breakthrough

The contribution of AMT, OSC, and RBV to the suppression of resistance under selective pressure from double combinations and the TCAD regimen was determined by passaging the virus five times in MDCK cells in the presence of double combinations, with increasing concentrations of a third drug titrated into the double combinations. Briefly, MDCK cells plated in 96-well microtiter plates (8×10^4^ cells/well) were incubated with influenza virus and a double combination of 0.30 µg/mL OSC and 0.60 µg/mL RBV, 0.6 µg/mL AMT and 0.6 µg/mL RBV, or 0.6 µg/mL AMT and 0.3 mg/mL OSC. These concentrations represent the average plasma concentrations for AMT, OSC, and RBV based on the doses of each drug (75 mg AMT three times day, 75 mg oseltamivir phosphate three times a day, and 200 mg RBV three times a day) used in a pilot Phase 1b study of TCAD therapy in immunocompromised patients (manuscript in preparation). The third drug was titrated as follows: AMT was titrated at 0, 0.1, 0.3, 0.6, 0.9, and 1.2 µg/mL into the OSC/RBV double combination, OSC was titrated at 0, 0.075, 0.15, 0.3, 0.6, and 1.2 µg/mL into the AMT/RBV double combination, and RBV was titrated at 0, 0.3, 0.6, 0.9, 1.2, and 2.4 µg/mL into the AMT/OSC double combination. Each condition was tested in 12 replicates. After 3 days incubation, 1 or 10 µL of the supernatant from each well was transferred to a fresh well of uninfected MDCK cells and incubated for an additional 3 days in the presence of drugs. This process was repeated for a total of 5 passages. After the 5th passage, the virus induced CPE in each well was measured by neutral red staining. Virus breakthrough was defined as wells having greater than 50% CPE. Sanger sequence analysis of the M2, HA, and NA genes was performed on the supernatant of each well having greater than 50% CPE to detect the presence of resistance-associated substitutions.

### Quantitative Allele-Specific Real-Time PCR (ASPCR)

Quantitative ASPCR (qASPCR) was performed in 10 µl reaction volumes in PRISM™ 384-well Clear Optical Reaction Plates (Applied Biosystems, Foster City, CA) as previously described [43]. Briefly, in each 10 µl reaction, 1 µl of template was added to 9 µl of qPCR reaction mix containing 900 nM of each Forward and Reverse primer 225 nM of the appropriate TaqMan® MGB probe, 1× TaqMan Universal PCR Master Mix (Applied Biosystems), and molecular-grade water (Promega Corp. Madison, WI). Separate reactions were carried out with either the wild-type or the mutant allele-specific primer (sequences for primers and probes are provided in [43]). Amplification and real-time fluorescence detections were performed on the 7900HT Real Time PCR System (Applied Biosystems) using the following PCR conditions: 3 min at 50°C for UNG treatment, 10 min at 95°C for Taq Polymerase activation, 40 cycles 15 s at 95°C for denaturation and 1 min at 60°C for annealing and extension. Using a constant threshold of 0.2 and an automatic baseline, a Ct value was obtained for each reaction in the Sequence Detection Systems v2.3 software (Applied Biosystems). Percentages of mutation present in the population were calculated from the delta Ct value using plasmids containing an insert of wild-type or mutant DNA sequence-allele plasmid templates as standards. Each well was initially analyzed by all ASPCR assays (V27A, A30T, and S31N for M2) as a single reaction. If resistant virus variants were detected at ≥1% of the population in more than one passage for a given condition (drug regimen, concentration, and MOI), then ASPCR analysis was repeated using triplicate reactions for all passages for that condition for more accurate quantification.

### Sanger sequence analysis

Sanger sequencing was performed on regions of the M2, HA, and NA genes containing previously identified antiviral resistance-conferring single nucleotide mutations. Sequence analysis was restricted to progeny derived from A/Hawaii/31/2007 (GenBank accession numbers EU516078.1, EU516139.1, and EU516164.1). No new sequence data were generated as a result of this study. Custom primer pairs targeting the NA, HA, and M2 genes were used for the PCR and cycle sequencing. Initial PCR reactions with 1 µl cDNA template in 14 µl master mix composed of 1× Taq Polymerase PCR Buffer (Invitrogen), 3 mM MgCl, 3 µM dNTP's, 200 nM primers, and 1 U Platinum Taq Polymerase (Invitrogen). PCR conditions on Biorad's DNA Engine Thermocycler were initial denaturation of 95°C for 5 minutes, followed by 35 cycles of: 95°C for 1 minute, 59°C for 30 seconds, 72°C for 1 minute, and a final extension of 72°C for 7 minute.

Applied Biosystems' Big Dye Terminator Kit v3.1 and 2.0 µM primers (same primers as initial PCR) were used in 10 µl cycle sequencing reactions consisting of 1 µl DNA template in 9 µl mastermix. Cycle sequencing was executed as: 96°C for 1 minute followed by 15 cycles of: 96°C for 10 seconds, 50°C for 5 seconds, 60°C for 1 minute 15 seconds, then 5 cycles of: 96°C for 10 seconds, 50°C for 5 seconds, 60°C for 1 minute 30 seconds, and ending with 5 cycles of: 96°C for 10 seconds, 50°C for 5 seconds, 60°C for 2 minute. Qiagen's DyeEx 96 kit was used for dye removal and samples were analyzed on Applied Biosystems' 3130xl Genetic Analyzer (36 cm array, Pop7 polymer). Sequences were analyzed for mutations at the codons of interest in Applied Biosystems' Sequencing Analysis v5.2 and DNASTAR Lasergene v8.0.

The sequences of the primers used are provided below (5′→3′), where Y represents a pyrimidine base (C or T), R represents a purine base (A or G), and M represents either A or C:

M2: Forward – CYAGCACTACAGCTAAGGCTATGGAGCA

 Reverse – CATCCACAGCAYTCTGCTGTTCCT

NA: Forward – CTGGAAGTCAAAACMACACTGGAATATGC

 Reverse – CTCCATCAACAGTCACTGGATTRCAGC

HA: Forward – GAGAATGGAACATGTTACCCAGGG

 Reverse – CTGATCCAAAGCCTCTACTCAGTGC

### Phenotype Analysis of Supernatants and Virus Clones

Susceptibility to antiviral drugs was determined as previously reported [21], [22]. Briefly, MDCK cells plated in 96-well microtiter plates (8×10^4^ cells/well) were incubated with influenza virus and either AMT (0.00032, 0.001, 0.0032, 0.01, 0.032, 0.1, 0.32, 1.0, 3.2, 10, 32, 100 µg/mL), OSC (0.001, 0.0032, 0.01, 0.032, 0.1, 0.32, 1.0, 3.2, 10, 32, 100, 320 µg/mL), ZAN (0.001, 0.0032, 0.01, 0.032, 0.1, 0.32, 1.0, 3.2, 10, 32, 100, 320 µg/mL), or RBV (0.001, 0.0032, 0.01, 0.032, 0.1, 0.32, 1.0, 3.2, 10, 32, 100, 320 µg/mL) for three days. Virus-induced CPE was determined by measuring cell viability by staining with neutral red dye and measuring the optical density at 540 nm. The spectrophotometric readings were collected electronically and imported into GraphPad Prism 5 software for four-parameter curve fit analysis. Two replicate plates were run on two separate days, with 3 replicate wells on each plate, and the data for each plate are presented along with the mean of all four plates.

## Supporting Information

Table S1 (DOC)

Table S2 (DOC)

Table S3 (DOC)
