# Supplementary material for: Triple Combination Antiviral Drug (TCAD) Composed of Amantadine, Oseltamivir, and Ribavirin Impedes the Selection of Drug-Resistant Influenza A Virus
Source: PLoS One. 2011 Dec 29;6(12):e29778. doi: 10.1371/journal.pone.0029778 (PMC3248427; doi:10.1371/journal.pone.0029778)
Supplement: Table S1 — (DOC) [file pone.0029778.s001.doc]

Table S1: Percent of virus variants with M2 (V27A, A30T, or S31N) substitutions as determined by qASPCR from serial passage at fixed concentrations

| **MOI** | **Regimen**  **(Conc.)** | **M2: V27A (% A)** | | | | | **M2: A30T (% T)** | | | | | **M2: S31N (% N)** | | | | | **Viral Load (log_10_ RNA copies/mL)** | | | | |
| --- | --- | --- | --- | --- | --- | --- | --- | --- | --- | --- | --- | --- | --- | --- | --- | --- | --- | --- | --- | --- | --- |
|  |  | P1 | P2 | P3 | P4 | P5 | P1 | P2 | P3 | P4 | P5 | P1 | P2 | P3 | P4 | P5 | P1 | P2 | P3 | P4 | P5 |
| 0.1 | Placebo | **0.3** | **0.3** | **0.0** | **0.1** | **0.3** | **0.0** | **0.0** | **0.1** | **0.1** | **0.0** | **0.0** | **0.0** | **0.1** | **0.0** | **0.0** | 9.1 | 8.1 | 8.2 | 8.5 | 7.9 |
| 0.01 | Placebo | **0.3** | **0.2** | **0.0** | **0.2** | **0.1** | **0.1** | **0.0** | **0.0** | **0.0** | **0.0** | **0.0** | **0.0** | **0.0** | **0.0** | **0.0** | 9.1 | 8.7 | 8.8 | 9.4 | 9.1 |
| 0.001 | Placebo | **0.3** | **0.2** | **0.1** | **0.2** | **0.2** | **0.1** | **0.0** | **0.0** | **0.0** | **0.0** | **0.0** | **0.0** | **0.0** | **0.0** | **0.0** | 9.1 | 8.4 | 8.6 | 9.0 | 8.6 |
| 0.1 | AMT (1) | 0.0 | 0.0 | 0.0 | 0.0 | 0.0 | 0.0 | 0.0 | 0.0 | 0.0 | 0.0 | 0.0 | 0.0 | 0.0 | 0.0 | 0.0 | 9.1 | 8.5 | 8.3 | 9.1 | 9.3 |
| 0.01 | AMT (1) | **0.3** | **0.2** | **0.0** | **4.2** | **10.7** | **0.0** | **0.0** | **0.0** | **0.0** | **0.0** | **0.0** | **0.0** | **0.0** | **0.0** | **0.0** | 8.9 | 8.8 | 8.3 | 9.3 | 9.1 |
| 0.001 | AMT (1) | 0.0 | 0.0 | 0.0 | 0.0 | 0.0 | 0.0 | 0.0 | 0.0 | 0.0 | 0.0 | 0.0 | 0.0 | 0.0 | 0.0 | 0.0 | 8.8 | 8.7 | 7.3 | 9.1 | 9.5 |
| 0.1 | AMT (2) | **0.3** | **2.2** | **51.9** | **68.7** | **89.9** | **0.0** | **0.0** | **0.0** | **0.0** | **0.0** | **0.0** | **0.0** | **0.0** | **0.2** | **0.0** | 8.9 | 7.6 | 6 | 8.6 | 6.2 |
| 0.01 | AMT (2) | **0.0** | **0.0** | **0.0** | **0.0** | **0.0** | 0.0 | 0.0 | 0.0 | 0.0 | 0.0 | 0.0 | 0.0 | 0.0 | 0.0 | 0.0 | 8.8 | 7.8 | 6.8 | 9.0 | 8.5 |
| 0.001 | AMT (2) | 0.0 | 0.0 | 0.0 | 0.0 | 0.0 | 1.9 | 0.0 | 0.0 | 0.0 | 0.0 | 0.0 | 0.0 | 0.0 | 0.0 | 0.0 | 8.8 | 8.2 | 7.0 | 9.0 | 8.3 |
| 0.1 | AMT (3) | **0.4** | **17.7** | **45.3** | **80.7** | **85.4** | **0.0** | **0.0** | **0.0** | **0.0** | **0.0** | **1.2** | **32.7** | **17.2** | **7.4** | **2.6** | 8.8 | 8.7 | 8.1 | 9.0 | 8.8 |
| 0.01 | AMT (3) | 0.0 | 0.0 | 0.0 | 0.0 | 1.6 | **0.0** | **0.0** | **0.0** | **0.0** | **0.0** | **0.5** | **60.2** | **98.1** | **97.5** | **98.3** | 8.8 | 7.7 | 7.9 | 9.0 | 9.2 |
| 0.001 | AMT (3) | 0.0 | 0.0 | 0.0 | 0.0 | 0.0 | 0.0 | 0.0 | 0.0 | 2.4 | 2.1 | 0.0 | 0.0 | 0.0 | 0.0 | 0.0 | 8.4 | 8.6 | 8.0 | 9.1 | 9.5 |
| 0.1 | AMT (4) | **0.5** | **30.7** | **56.0** | **72.4** | **82.5** | **0.0** | **0.0** | **0.0** | **0.0** | **0.0** | **0.0** | **0.3** | **0.0** | **0.0** | **0.0** | 8.6 | 8.5 | 6.7 | 8.7 | 7.4 |
| 0.01 | AMT (4) | **0.5** | **78.8** | **99.3** | **99.9** | **99.4** | **0.0** | **0.0** | **0.0** | **0.2** | **0.0** | **0.8** | **0.0** | **0.0** | **0.0** | **0.0** | 8.5 | 8 | 8.0 | 8.4 | 6.0 |
| 0.001 | AMT (4) | 0.0 | 0.0 | 0.0 | 0.0 | 0.0 | 0.0 | 0.0 | 0.0 | 0.0 | 0.0 | 0.0 | 0.0 | 0.0 | 0.0 | 0.0 | 7.0 | 2.8 | 3.9 | 8.5 | 8.5 |
| 0.1 | OSC (1) | 0.0 | 0.0 | 0.0 | 0.0 | 0.0 | 0.0 | 0.0 | 0.0 | 0.0 | 0.0 | 0.0 | 0.0 | 0.0 | 0.0 | 0.0 | 9.0 | 8.6 | 8.2 | 9.0 | 8.9 |
| 0.01 | OSC (1) | 0.0 | 0.0 | 0.0 | 0.0 | 0.0 | 0.0 | 0.0 | 0.0 | 0.0 | 0.0 | 0.0 | 0.0 | 0.0 | 0.0 | 0.0 | 9.1 | 8.6 | 8.6 | 8.9 | 9.1 |
| 0.001 | OSC (1) | 0.0 | 0.0 | 0.0 | 0.0 | 0.0 | 0.0 | 0.0 | 0.0 | 0.0 | 0.0 | 0.0 | 0.0 | 0.0 | 0.0 | 0.0 | 9.2 | 8 | 8.3 | 8.8 | 7.9 |
| 0.1 | OSC (2) | 0.0 | 0.0 | 0.0 | 0.0 | 0.0 | 0.0 | 0.0 | 0.0 | 0.0 | 0.0 | 0.0 | 0.0 | 0.0 | 0.0 | 0.0 | 9.0 | 7.9 | 8.3 | 8.9 | 9.0 |
| 0.01 | OSC (2) | 0.0 | 0.0 | 0.0 | 0.0 | 0.0 | 0.0 | 0.0 | 1.7 | 0.0 | 0.0 | 0.0 | 0.0 | 0.0 | 0.0 | 0.0 | 9.1 | 8.1 | 7.8 | 9.1 | 9.4 |
| 0.001 | OSC (2) | 0.0 | 0.0 | 0.0 | 0.0 | 0.0 | 0.0 | 0.0 | 0.0 | 0.0 | 0.0 | 0.0 | 0.0 | 0.0 | 0.0 | 0.0 | 9.1 | 8.2 | 7.8 | 9.1 | 9.3 |
| 0.1 | OSC (3) | 0.0 | 0.0 | 0.0 | 0.0 | 0.0 | 0.0 | 0.0 | 0.0 | 0.0 | 0.0 | 0.0 | 0.0 | 0.0 | 0.0 | 0.0 | 8.6 | 8.3 | 8.8 | 9.2 | 9.4 |
| 0.01 | OSC (3) | 0.0 | 0.0 | 0.0 | 0.0 | 0.0 | 0.0 | 0.0 | 0.0 | 0.0 | 0.0 | 0.0 | 0.0 | 0.0 | 0.0 | 0.0 | 8.7 | 8.1 | 8.4 | 9.2 | 9.0 |
| 0.001 | OSC (3) | 0.0 | 0.0 | 0.0 | 0.0 | 0.0 | 0.0 | 0.0 | 0.0 | 0.0 | 0.0 | 0.0 | 0.0 | 0.0 | 0.0 | 0.0 | 9.0 | 8.0 | 8.9 | 3.9 | 4.7 |
| 0.1 | OSC (4) | 0.0 | 0.0 | 0.0 | 0.0 | 0.0 | 0.0 | 0.0 | 0.0 | 0.0 | 0.0 | 0.0 | 0.0 | 0.0 | 0.0 | 0.0 | 8.9 | 7.8 | 8.5 | 9.2 | 9.2 |
| 0.01 | OSC (4) | 0.0 | 0.0 | 0.0 | 0.0 | 0.0 | 0.0 | 0.0 | 0.0 | 0.0 | 0.0 | 0.0 | 0.0 | 0.0 | 0.0 | 0.0 | 8.9 | 8.5 | 7.1 | 9.3 | 9.4 |
| 0.001 | OSC (4) | 0.0 | 0.0 | 0.0 | 0.0 | 0.0 | 0.0 | 0.0 | 0.0 | 0.0 | 1.2 | 0.0 | 0.0 | 0.0 | 0.0 | 0.0 | 8.6 | 7.9 | 8.0 | 4.2 | 4.4 |

Wild type influenza A/Hawaii/31/2007 (H1N1) virus was passaged five times in MDCK cells, with the concentrations of drugs in each regimen kept fixed in between passages. The percent of virus variants bearing resistance-associated substitutions in M2 (V27A, A30T, or S31N) are presented. Unbolded values are from single qASPCR reactions, whereas bolded values are the mean of triplicate qASPCR reactions. NA = no amplification detected.

Table S1: Percent of virus variants with M2 (V27A, A30T, or S31N) substitutions as determined by ASPCR from serial passage at fixed concentrations (continued)

| **MOI** | **Regimen**  **(Conc.)** | **ASPCR (M2: V27A) - % A** | | | | | **ASPCR (M2: A30T) - % T** | | | | | **ASPCR (M2: S31N) - % N** | | | | | **Viral Load (log_10_ RNA copies/mL)** | | | | |
| --- | --- | --- | --- | --- | --- | --- | --- | --- | --- | --- | --- | --- | --- | --- | --- | --- | --- | --- | --- | --- | --- |
|  |  | P1 | P2 | P3 | P4 | P5 | P1 | P2 | P3 | P4 | P5 | P1 | P2 | P3 | P4 | P5 | P1 | P2 | P3 | P4 | P5 |
| 0.1 | AMT/OSC (1) | 0.0 | 0.0 | 0.0 | 1.9 | 0.0 | 0.0 | 0.0 | 0.0 | 0.0 | 0.0 | 0.0 | 0.0 | 0.0 | 0.0 | 0.0 | 9.0 | 7.8 | 4.6 | 9.0 | 9.2 |
| 0.01 | AMT/OSC (1) | 0.0 | 0.0 | 0.0 | 0.0 | 0.0 | 0.0 | 0.0 | 0.0 | 0.0 | 0.0 | 0.0 | 0.0 | 0.0 | 0.0 | 0.0 | 9.1 | 8.8 | 5.6 | 8.9 | 9.3 |
| 0.001 | AMT/OSC (1) | 0.0 | 0.0 | 0.0 | 0.0 | 0.0 | 0.0 | 0.0 | 0.0 | 0.0 | 0.0 | 0.0 | 0.0 | 0.0 | 0.0 | 0.0 | 9.1 | 8.6 | 3.8 | 9.3 | 8.5 |
| 0.1 | AMT/OSC (2) | 0.0 | 0.0 | 0.0 | 0.0 | 2.4 | **0.0** | **0.0** | **2.1** | **2.7** | **2.3** | 0.0 | 0.0 | 0.0 | 0.0 | 0.0 | 8.8 | 8.1 | 8.6 | 8.6 | 9.1 |
| 0.01 | AMT/OSC (2) | **0.3** | **6.6** | **51.8** | **77.8** | **92.8** | **0.0** | **0.0** | **0.0** | **0.0** | **0.0** | **0.0** | **0.0** | **0.0** | **0.0** | **0.0** | 8.7 | 8.6 | 8.6 | 8.5 | 9.5 |
| 0.001 | AMT/OSC (2) | 0.0 | 0.0 | 0.0 | 0.0 | 0.0 | **0.0** | **0.0** | **0.0** | **0.0** | **0.0** | **0.0** | **0.0** | **0.0** | **0.0** | **0.0** | 8.0 | 6.0 | 3.4 | 8.7 | 7.9 |
| 0.1 | AMT/OSC (3) | **0.8** | **74.3** | **99.6** | **100.0** | **100.0** | **0.2** | **0.0** | **0.0** | **0.0** | **0.0** | **0.0** | **0.0** | **0.0** | **0.0** | **0.0** | 8.6 | 7.6 | 4.9 | 9.0 | 8.1 |
| 0.01 | AMT/OSC (3) | 0.0 | 0.0 | 0.0 | 0.0 | 0.0 | 0.0 | 0.0 | 0.0 | 0.0 | 0.0 | 0.0 | 0.0 | 0.0 | 0.0 | 0.0 | 8.1 | 4.8 | 2.5 | 3.6 | 3.1 |
| 0.001 | AMT/OSC (3) | **0.0** | **0.0** | **0.0** | **0.0** | NA | **0.0** | **0.0** | **0.0** | **0.0** | **0.0** | **0.0** | **0.0** | **0.0** | **0.0** | **0.0** | 7.0 | 5.3 | 2.6 | 2.8 | 4.5 |
| 0.1 | AMT/OSC (4) | **1.4** | **0.0** | **0.0** | **0.0** | **0.0** | **0.0** | **0.0** | **0.0** | **0.0** | **0.0** | **0.0** | **0.4** | **0.0** | **0.0** | **0.0** | 8.2 | 6.2 | 5.3 | 6.0 | 2.8 |
| 0.01 | AMT/OSC (4) | 0.0 | 3.7 | 0.0 | 0.0 | 7.1 | 0.0 | 0.0 | 0.0 | 0.0 | 0.0 | 0.0 | 0.0 | 0.0 | 0.0 | 0.0 | 7.5 | 5.6 | 5.6 | NA | 4.6 |
| 0.001 | AMT/OSC (4) | 0.0 | 0.0 | 0.0 | 0.0 | 8.2 | 0.0 | 0.0 | 0.0 | 0.0 | 0.0 | 0.0 | 0.0 | 0.0 | 0.0 | 0.0 | 5.7 | 4.3 | 4.1 | 6.3 | 5.7 |
| 0.1 | TCAD (1) | **0.5** | **1.1** | **3.5** | **8.0** | **29.6** | **0.0** | **0.0** | **0.0** | **0.0** | **0.0** | 0.0 | 0.0 | 0.0 | 0.0 | 0.0 | 9.1 | 8.1 | 8.1 | 5.5 | 8.4 |
| 0.01 | TCAD (1) | 0.0 | 0.0 | 0.0 | 0.0 | 0.0 | 0.0 | 0.0 | 0.0 | 0.0 | 0.0 | 0.0 | 0.0 | 0.0 | 0.0 | 0.0 | 8.9 | 8.6 | 8.3 | 8.2 | 9.2 |
| 0.001 | TCAD (1) | 0.0 | 0.0 | 0.0 | 0.0 | 0.0 | 0.0 | 0.0 | 0.0 | 0.0 | 0.0 | 0.0 | 0.0 | 0.0 | 0.0 | 0.0 | 9.0 | 8.5 | 7.1 | NA | 4.3 |
| 0.1 | TCAD (2) | **0.2** | **0.0** | **0.1** | **0.2** | **0.1** | **0.0** | **5.1** | **10.9** | **21.5** | **27.5** | **0.0** | **0.0** | **0.0** | **0.0** | **0.0** | 9.0 | 7.3 | 8.5 | 8.8 | 6.9 |
| 0.01 | TCAD (2) | 0.0 | 0.0 | 0.0 | 0.0 | 0.0 | 0.0 | 0.0 | 0.8 | 0.0 | 0.0 | 0.0 | 0.0 | 0.0 | 0.0 | 0.0 | 8.7 | 8.8 | 8.4 | 9.2 | 8.6 |
| 0.001 | TCAD (2) | **0.2** | **0.0** | **0.0** | **0.0** | **0.0** | **0.0** | **0.0** | **0.0** | **0.0** | **0.0** | **0.0** | **0.0** | **0.0** | **0.0** | **0.0** | 7.9 | 6.9 | 5.5 | 9.0 | 8.9 |
| 0.1 | TCAD (3) | **0.0** | **0.0** | **0.0** | **1.1** | **0.0** | **0.1** | **0.0** | **0.0** | **0.0** | **0.0** | **0.4** | **0.0** | **0.0** | **0.0** | **0.0** | 8.2 | 6.8 | 5.2 | NA | 4.6 |
| 0.01 | TCAD (3) | 0.0 | 0.0 | 0.0 | 0.0 | 0.0 | 0.0 | 0.0 | 0.0 | 0.0 | 0.0 | 2.5 | 0.0 | 0.0 | 0.0 | 0.0 | 7.7 | 3.5 | 2.8 | 4.7 | 5.5 |
| 0.001 | TCAD (3) | **0.0** | **0.0** | **0.0** | **0.0** | **0.0** | **0.3** | **27.1** | **25.6** | **47.4** | **35.0** | **0.0** | **0.0** | **0.0** | **0.0** | **0.0** | 6.2 | 5.8 | 5.1 | 2.1 | 4.7 |
| 0.1 | TCAD (4) | 0.0 | 0.0 | 0.0 | 0.0 | 0.0 | 0.0 | 0.0 | 0.0 | 0.0 | 0.0 | 0.0 | 0.0 | 0.0 | 0.0 | 0.0 | 6.2 | 4.2 | 3.6 | 2.5 | 4.6 |
| 0.01 | TCAD (4) | **0.0** | **19.4** | **0.0** | **0.0** | **0.0** | **0.1** | **0.0** | **0.0** | **0.0** | **0.0** | **0.0** | **0.0** | **0.0** | **0.0** | **0.0** | 5.1 | 4.0 | 2.9 | 4.0 | 5.0 |
| 0.001 | TCAD (4) | 0.0 | 0.0 | 0.0 | 0.0 | 2.1 | 0.0 | 0.0 | 0.0 | 0.0 | 0.0 | 0.0 | 0.0 | 0.0 | 0.0 | 0.0 | 3.2 | 3.2 | 0.7 | 2.3 | 4.6 |

Wild type influenza A/Hawaii/31/2007 (H1N1) virus was passaged five times in MDCK cells, with the concentrations of drugs in each regimen kept fixed in between passages. The percent of virus variants bearing resistance-associated substitutions in M2 (V27A, A30T, or S31N) are presented. Unbolded values are from single qASPCR reactions, whereas bolded values are the mean of triplicate qASPCR reactions. NA = no amplification detected.
