# Supplementary material for: Triple Combination Antiviral Drug (TCAD) Composed of Amantadine, Oseltamivir, and Ribavirin Impedes the Selection of Drug-Resistant Influenza A Virus
Source: PLoS One. 2011 Dec 29;6(12):e29778. doi: 10.1371/journal.pone.0029778 (PMC3248427; doi:10.1371/journal.pone.0029778)
Supplement: Table S2 — (DOC) [file pone.0029778.s002.doc]

Table S2: Amino Acid Identity of M2 and HA Genes by Sanger Sequence Analysis from Serial Passage at Fixed Concentrations^1^

| **Condition** | | | | **M2 (Sanger Method)** | | | | | **M2**  **(qASPCR)** | **HA (Sanger Method)** | |
| --- | --- | --- | --- | --- | --- | --- | --- | --- | --- | --- | --- |
| Regimen | Concentration | MOI | Passage | L26 | V27 | A30 | S31 | G34 |  | N163 | S165 |
| AMT | 2 | 0.1 | 5 | not tested | | | | | V27A |  |  |
| AMT | 2 | 0.01 | 5 | not tested | | | | |  |  |  |
| AMT | 3 | 0.1 | 5 |  | A |  |  |  | V27A |  |  |
| AMT | 3 | 0.01 | 5 |  |  |  | S/N* |  | S31N |  |  |
| AMT | 4 | 0.1 | 5 |  | V/A* |  |  | G/E* | V27A |  | S/R* |
| AMT | 4 | 0.01 | 5 |  | A |  |  |  | V27A |  | S/R* |
| OSC | 1 | 0.1 | 5 | not tested | | | | |  | N/T* | R |
| OSC | 1 | 0.01 | 5 | not tested | | | | |  |  |  |
| OSC | 2 | 0.1 | 5 | not tested | | | | |  | N/T* |  |
| OSC | 2 | 0.01 | 5 | not tested | | | | |  | N/T* |  |
| OSC | 3 | 0.1 | 5 |  |  |  |  |  |  |  |  |
| OSC | 3 | 0.01 | 5 |  |  |  |  |  |  |  |  |
| OSC | 4 | 0.1 | 5 |  |  |  |  |  |  | T |  |
| OSC | 4 | 0.01 | 5 |  |  |  |  |  |  | N/T* |  |
| AMT/OSC | 1 | 0.1 | 5 |  |  |  |  |  |  | N/T* | S/R* |
| AMT/OSC | 1 | 0.01 | 5 |  |  |  |  |  |  | N/T* | S/R* |
| AMT/OSC | 2 | 0.1 | 5 | not tested | | | | |  | T |  |
| AMT/OSC | 2 | 0.01 | 5 | not tested | | | | |  |  | R |
| AMT/OSC | 3 | 0.1 | 5 |  | V/A* |  |  |  | V27A |  | S/R* |
| TCAD | 1 | 0.1 | 5 | L/F* |  |  |  |  | V27A |  |  |
| TCAD | 1 | 0.01 | 5 |  |  |  |  |  |  | N/T* | S/R* |
| TCAD | 2 | 0.1 | 5 |  |  | A/V* |  |  | A30T |  |  |
| TCAD | 3 | 0.1 | 5 | failed amplification | | | | |  | failed amplification | |
| TCAD | 3 | 0.01 | 5 | failed amplification | | | | |  | failed amplification | |
| TCAD | 3 | 0.001 | 5 | failed amplification | | | | |  | failed amplification | |

Wild type influenza A/Hawaii/31/2007 (H1N1) virus was passaged five times in MDCK cells, with the concentrations of drugs in each regimen kept fixed in between passages. All conditions were tested by qASPCR after each passage, whereas selected conditions were tested by Sanger sequence analysis after the last passage. NA was also sequenced though no substitutions were detected. ^1^All amino acid substitutions in the M2 and HA gene after the last passage relative to the input virus sequence are reported as described in methods. Blank indicates no change. qASPCR results for M2 are provided for comparison. *Indicates mixed population (2 visible peaks).
