# Supplementary material for: Triple Combination Antiviral Drug (TCAD) Composed of Amantadine, Oseltamivir, and Ribavirin Impedes the Selection of Drug-Resistant Influenza A Virus
Source: PLoS One. 2011 Dec 29;6(12):e29778. doi: 10.1371/journal.pone.0029778 (PMC3248427; doi:10.1371/journal.pone.0029778)
Supplement: Table S3 — (DOC) [file pone.0029778.s003.doc]

Table S3: Passage History for Serial Passage at Increasing Concentrations

| Regimen | Passage | Cumulative days in culture | Concentration* (μg/mL) | Fold Increase | M2 Sequence† | | | | | Neuraminidase Sequence† | | | | | Hemagglutinin Sequence† | | | |
| --- | --- | --- | --- | --- | --- | --- | --- | --- | --- | --- | --- | --- | --- | --- | --- | --- | --- | --- |
|  |  |  |  |  | L-26 | V-27 | A-30 | S-31 | G-34 | E-119 | R-152 | H-274 | R-292 | N-294 | N-163 | S-165 | Y-168 | D-190 |
| No Drug Control | P7 | 19 | 0 | N/A |  |  |  |  |  |  |  |  |  |  |  |  |  | N |
| AMT | P1 | 3 | 0.015 | 1 | ND | | | | | ND | | | | | ND | | | |
|  | P2 | 6 | 0.06 | 4 | ND | | | | | ND | | | | | ND | | | |
|  | P3 | 10 | 0.24 | 16 |  |  |  |  |  | ND | | | | | ND | | | |
|  | P4 | 13 | 0.96 | 64 | F |  |  |  |  | ND | | | | | ND | | | |
|  | P5 | 16 | 3.84 | 256 | F |  |  |  |  | ND | | | | | ND | | | |
|  | P6 | 19 | 15.36 | 1024 | F |  |  |  |  | ND | | | | | ND | | | |
|  | **P7** | 22 | 33 | 2200 | F |  |  |  |  | ND | | | | | ND | | | |
| OSC | P1 | 3 | 0.03 | 1 | ND | | | | | ND | | | | |  |  |  |  |
|  | P2 | 6 | 0.12 | 4 | ND | | | | | ND | | | | |  | R |  |  |
|  | P3 | 9 | 0.48 | 16 | ND | | | | |  |  |  |  |  |  | R |  |  |
|  | P4 | 12 | 1.92 | 64 | ND | | | | | ND | | | | | ND | | | |
|  | P5 | 15 | 7.68 | 256 | ND | | | | | ND | | | | | ND | | | |
|  | P6 | 19 | 15.36 | 512 | ND | | | | | ND | | | | | ND | | | |
|  | **P7** | 25 | 30.72 | 1024 | ND | | | | |  |  |  |  |  |  | R |  |  |
| ZAN | P1 | 3 | 0.03 | 1 | ND | | | | | ND | | | | |  |  |  |  |
|  | P2 | 6 | 0.12 | 4 | ND | | | | | ND | | | | |  |  |  |  |
|  | P3 | 9 | 0.48 | 16 | ND | | | | |  |  |  |  |  |  | R |  |  |
|  | P4 | 12 | 1.92 | 64 | ND | | | | | ND | | | | | ND | | | |
|  | P5 | 15 | 7.68 | 256 | ND | | | | | ND | | | | | ND | | | |
|  | P6 | 19 | 30.72 | 1024 | ND | | | | | ND | | | | | ND | | | |
|  | **P7** | 22 | 118 | 3933 | ND | | | | |  |  |  |  |  |  | R |  |  |

*Concentrations of drugs for combinations are provided in order of drugs as listed in the first column. †Amino acid positions for M2 and neuraminidase are listed for which substitutions have been shown to confer resistance to adamantanes or oseltamivir, respectively. Amino acid positions for hemagglutinin are listed for which substitutions occurred relative to the input virus sequence. The input virus amino acid sequence is shown in the column heading using the one letter abbreviation. Changes from the input virus sequence are indicated (blanks indicate no change). Amino acid positions for NA and HA are based on N2 and H3 numbering. Passages in bold indicate phenotypic analysis was performed on the supernatant. ND – not determined. Fail – no PCR amplification.

Table S3: Passage History for Serial Passage at Increasing Concentrations (continued)

| Regimen | Passage | Cumulative days in culture | Concentration* (mcg/mL) | Fold Increase | M2 Sequence† | | | | | Neuraminidase Sequence† | | | | | Hemagglutinin Sequence† | | | |
| --- | --- | --- | --- | --- | --- | --- | --- | --- | --- | --- | --- | --- | --- | --- | --- | --- | --- | --- |
|  |  |  |  |  | L-26 | V-27 | A-30 | S-31 | G-34 | E-119 | R-152 | H-274 | R-292 | N-294 | N-163 | S-165 | Y-168 | D-190 |
| RBV | P1 | 3 | 1.5 | 1 | ND | | | | | ND | | | | | ND | | | |
|  | P2 | 7 | 6 | 4 | ND | | | | | ND | | | | | ND | | | |
|  | **P3** | 13 | 24 | 16 |  |  |  |  |  |  |  |  |  |  |  |  |  |  |
|  | P4 | 19 | 96 | 64 | ND | | | | | ND | | | | | ND | | | |
|  | P5 | 22 | 96 | 64 | ND | | | | | ND | | | | | ND | | | |
|  | P6 | 28 | 96 | 64 | ND | | | | | ND | | | | | ND | | | |
|  | P7 | 34 | 96 | 64 | Fail | | | | | Fail | | | | |  |  |  |  |
| AMT/OSC | P1 | 3 | 0.015/0.03 | 1 |  |  |  |  |  | ND | | | | |  |  |  |  |
|  | P2 | 6 | 0.06/0.12 | 4 |  |  |  |  |  | ND | | | | |  |  |  |  |
|  | P3 | 9 | 0.24/0.48 | 16 |  |  |  |  |  |  |  |  |  |  |  |  |  |  |
|  | **P4** | 15 | 0.24/0.48 | 16 |  |  |  |  |  | ND | | | | |  | R |  |  |
|  | P5 | 21 | 0.24/0.48 | 16 | Fail | | | | |  |  |  |  |  |  | R | F |  |
|  | P6 | 27 | 0.24/0.48 | 16 | Fail | | | | | ND | | | | | Fail | | | |
| OSC/ZAN | P1 | 3 | 0.03/0.03 | 1 | ND | | | | | ND | | | | |  |  |  |  |
|  | P2 | 6 | 0.12/0.12 | 4 | ND | | | | | ND | | | | |  |  |  |  |
|  | P3 | 9 | 0.48/0.48 | 16 | ND | | | | |  |  |  |  |  |  | R |  |  |
|  | P4 | 12 | 1.92/1.92 | 64 | ND | | | | | ND | | | | | ND | | | |
|  | P5 | 16 | 7.68/7.68 | 256 | ND | | | | | ND | | | | | ND | | | |
|  | P6 | 20 | 7.68/7.68 | 256 | ND | | | | | ND | | | | | ND | | | |
|  | **P7** | 26 | 30.72/30.72 | 1024 | ND | | | | |  |  |  |  |  |  | R |  |  |
| AMT/OSC/RBV | P1 | 4 | 0.015/0.03/1.5 | 1 |  |  |  |  |  | ND | | | | |  |  |  |  |
|  | P2 | 9 | 0.03/0.06/3 | 2 |  |  |  |  |  | ND | | | | |  |  |  |  |
|  | **P3** | 13 | 0.03/0.06/3 | 2 |  |  |  |  |  |  |  |  |  |  | T |  |  |  |
|  | P4 | 19 | 0.03/0.06/3 | 2 |  |  |  |  |  | ND | | | | | T |  |  |  |
|  | P5 | 25 | 0.03/0.06/3 | 2 | Fail | | | | | Fail | | | | | Fail | | | |
|  | P6 | 31 | 0.03/0.06/3 | 2 | Fail | | | | | Fail | | | | | Fail | | | |

*Concentrations of drugs for combinations are provided in order of drugs as listed in the first column. †Amino acid positions for M2 and neuraminidase are listed for which substitutions have been shown to confer resistance to adamantanes or oseltamivir, respectively. Amino acid positions for hemagglutinin are listed for which substitutions occurred relative to the input virus sequence. The input virus amino acid sequence is shown in the column heading using the one letter abbreviation. Changes from the input virus sequence are indicated (blanks indicate no change). Amino acid positions for NA and HA are based on N2 and H3 numbering. Passages in bold indicate phenotypic analysis was performed on the supernatant. ND – not determined. Fail – no PCR amplification.
